# Labelling and targeted ablation of specific bipolar cell types in the zebrafish retina

**DOI:** 10.1186/1471-2202-10-107

**Published:** 2009-08-27

**Authors:** Xiao-Feng Zhao, Staale Ellingsen, Anders Fjose

**Affiliations:** 1 Department of Molecular Biology, University of Bergen, PO Box 7803, N-5020 Bergen, Norway; 2 Molecular and Behavioral Neuroscience Institute, University of Michigan, Ann Arbor, MI 48109, USA; 3 NIFES, National Institute of Nutrition and Seafood Research Strandgaten 229, N-5004 Bergen, Norway

## Abstract

**Background:**

Development of a functional retina depends on regulated differentiation of several types of neurons and generation of a highly complex network between the different types of neurons. In addition, each type of retinal neuron includes several distinct morphological types. Very little is known about the mechanisms responsible for generating this diversity of retinal neurons, which may also display specific patterns of regional distribution.

**Results:**

In a screen in zebrafish, using a trapping vector carrying an engineered yeast Gal4 transcription activator and a UAS:eGFP reporter cassette, we have identified two transgenic lines of zebrafish co-expressing eGFP and Gal4 in specific subsets of retinal bipolar cells. The eGFP-labelling facilitated analysis of axon terminals within the inner plexiform layer of the adult retina and showed that the fluorescent bipolar cells correspond to previously defined morphological types. Strong regional restriction of eGFP-positive bipolar cells to the central part of the retina surrounding the optic nerve was observed in adult zebrafish. Furthermore, we achieved specific ablation of the labelled bipolar cells in 5 days old larvae, using a bacterial nitroreductase gene under Gal4-UAS control in combination with the prodrug metronidazole. Following prodrug treatment, nitroreductase expressing bipolar cells were efficiently ablated without affecting surrounding retina architecture, and recovery occurred within a few days due to increased generation of new bipolar cells.

**Conclusion:**

This report shows that enhancer trapping can be applied to label distinct morphological types of bipolar cells in the zebrafish retina. The genetic labelling of these cells yielded co-expression of a modified Gal4 transcription activator and the fluorescent marker eGFP. Our work also demonstrates the potential utility of the Gal4-UAS system for induction of other transgenes, including a bacterial nitroreductase fusion gene, which can facilitate analysis of bipolar cell differentiation and how the retina recovers from specific ablation of these cells.

## Background

The vertebrate neural retina exhibits special features of cell differentiation, organization and synaptic connections that make it an excellent model for studying fundamental principles of neurobiology [1,2]. It consists of six major classes of neurons and one type of glia (Müller glia) that are generated from a common pool of retinal progenitor cells (RPCs). During development the different retinal cell types establish three nuclear layers; the innermost ganglion cell layer (GCL); amacrine, bipolar, horizontal, and Müller glial cells in the inner nuclear layer (INL); and rod and cone photoreceptors in the outer nuclear layer (ONL). The connectivity between the retinal neurons is mainly confined to two distinct synaptic layers, the inner plexiform layer (IPL) and outer plexiform layer (OPL), which separate the three nuclear layers. Additional complexity is due to the presence of multiple morphological types for each of the main classes of neurons and highly branched networks of synaptic connections [1].

Retinal neurons are generated from multipotent RPCs in a particular temporal order where the first post-mitotic cells differentiate as RGCs, followed by the other neuronal classes during partially overlapping time windows [2,3], and this has been suggested to reflect temporal changes in the competence states of the RPCs to produce subsets of cell types [4]. The competence states are intrinsically defined by specific combinations of transcription factors [5], and extrinsic signals that control the timing of RPC competence [6].

Bipolar neurons transmit signals from photoreceptors to the retinal ganglion cells (RGCs), and 17 different morphological types of these cells have been identified in zebrafish [7]. Synaptic connections of bipolar cells that are hyperpolarized or depolarized with increased light intensity are confined to the outer half (OFF sublamina) and inner half (ON sublamina) of the IPL, respectively [8,9]. Each of these sublaminae is composed of three functionally specialized sublayers [7]. Notably, this stratification is most clearly reflected in the axon terminal ramification patterns of the different types of bipolar cells [7].

Further investigations of differentiation, physiological functions and regeneration potentials of retinal neurons in zebrafish will depend on the availability of highly specific tools for in vivo visualization and manipulation of gene expression. In addition, it will be important to exploit novel techniques that can facilitate identification of the genes associated with these processes. Methods based on transposon or retroviral vectors have been established in zebrafish that provide opportunities for in vivo visualization and identification of new expression patterns through gene- or enhancer trapping [10-13]. The most recently developed techniques have combined the use of green fluorescent protein (GFP) reported enhancer/gene trapping with the flexibility of the Gal4-UAS system [14-16]. Hence, this novel technology allows for co-expression of various transgenes in the same fluorescently labelled cells. The aim of the work described in this report was to apply these trapping techniques to generate transgenic lines of zebrafish that co-express GFP and the transcriptional activator Gal4 in specific subsets of bipolar cells to establish new tools for studies of their differentiation and function.

## Results

### Two enhancer trap lines with Gal4-VP16 and eGFP expression in retinal cells

We used the Tol2-based transposon vector SAGVG (Gal4-VP16;UAS:eGFP), which was recently reported [16], to generate transgenic lines showing co-expression of eGFP (enhanced GFP) and the hybrid transcription factor Gal4-VP16 in specific retinal cells. Although the SAGVG construct was designed to function as a gene/enhancer trap, it mainly inserts and expresses Gal4-VP16/eGFP in a way that is consistent with an enhancer trap mechanism [16]. In this self-reporting vector, expression of eGFP is mediated by a 14 × UAS element in front of the basal promoter from the E1b gene [16].

**Figure 1:**
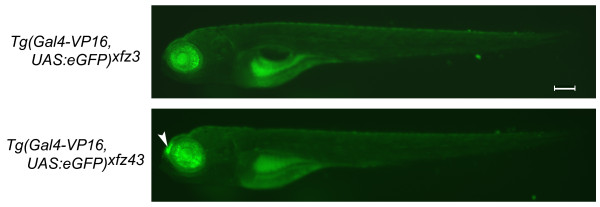
**Spatial patterns of eGFP expression in two different enhancer trap lines**. Fluorescence microscopy images of whole mount larvae at 5 dpf show eGFP expression restricted to the retina in both lines. The *Tg(Gal4-VP16;UAS:eGFP)*^*xfz*43 ^line has an additional site of expression in the olfactory placodes (arrowhead). Both lines show some auto-fluorescence in the yolk sac and gut. Scale bar: 0.2 mm.

From a small-scale screen performed in our laboratory [17], we identified two transgenic lines, *Tg(Gal4-VP16;UAS:eGFP)*^*xfz*3 ^and *Tg(Gal4-VP16;UAS:eGFP)*^*xfz*43^, referred to as *xfz3 *and *xfz43*, respectively, which showed reproducible expression in a subset of retinal cells (Figure 1). In addition to retina specific expression, the *xfz43 *line also displayed a high level of eGFP in the olfactory placodes of larvae (Figure 1).

The vector insertions associated with the eGFP expression of *xfz3 *and *xfz43 *were mapped to single sites on chromosome 2 and chromosome 6, respectively (Methods). Based on the current annotation of the zebrafish genome, neither of the two integrations were found to be located within a transcript, suggesting that these transgenic lines are enhancer traps. Furthermore, in situ hybridization analysis of the expression patterns of four candidate genes located closest to each of the insertions within regions of ~50 kb and ~400 kb in *xfz3 *and *xfz43 *(see Methods), respectively, did not show any similarity with respect to retinal eGFP expression in the transgenic lines (data not shown). Hence, the expression in the two transgenic lines appear to be controlled by enhancers for remote genes that we could not identify. However, we cannot exclude that genes may be found closer to these insertion sites in the future when annotation of the zebrafish genome is more complete.

### Retinal eGFP expression parallels onset of bipolar cell differentiation

**Figure 2:**
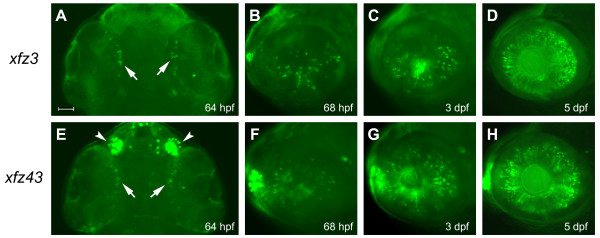
**Retinal expression of eGFP in enhancer trap lines at early larval stages**. Fluorescence microscopy of eGFP expression in the *Tg(Gal4-VP16;UAS:eGFP)*^*xfz*3 ^line (A-D). A ventral view (A) shows initial eGFP labelling of single cells (arrows) within the ventral-nasal region of the retina at 64 hpf. Lateral views (B-D), with anterior to the left, show increasing numbers of eGFP expressing retinal cells at 68 hpf (B), 3 dpf (C) and 5 dpf (D). (E-H) Expression of eGFP in the *Tg(Gal4-VP16;UAS:eGFP)*^*xfz*43 ^line. Ventral view (E) shows strong eGFP labelling in the olfactory placodes (arrowheads) and low levels of expression in single cells within the ventral-nasal region of the retina at 64 hpf (arrows). Lateral views (F-H), with anterior to the left, show increasing numbers of eGFP expressing retinal cells at 68 hpf (F), 3 dpf (G) and 5 dpf (H). Scale bar: 50 μm.

**Figure 3:**
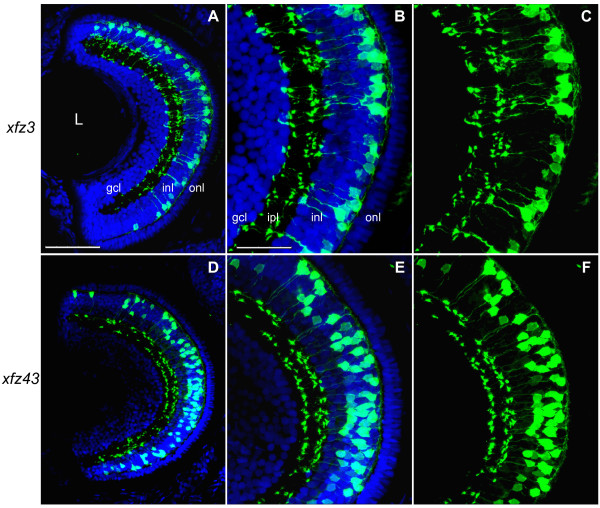
**Enhancer trap lines express eGFP in different types of bipolar cells in larvae**. Retinal cryosection of *Tg(Gal4-VP16;UAS:eGFP)*^*xfz*3 ^larva at 5 dpf (A-C). (A) Confocal image of cross-section (dorsal at the top) stained with an anti-GFP antibody (green) and counterstained with DAPI (blue). (B) Higher magnification image of the central part of the retina shown in (A). (C) Same confocal image as in (B) without DAPI. (D-F) Retinal cryosection from a *Tg(Gal4-VP16;UAS:eGFP)*^*xfz*43 ^larva at 5 dpf. The confocal images are arranged in the same way as shown for the other line. Scale bar: 50 μm (A, D); 25 μm (B, C, E, F). Abbreviations: gcl, ganglion cell layer; inl, inner nuclear layer; ipl, inner plexiform layer; L, lens; onl, outer nuclear layer.

In both transgenic lines, we first detected retinal eGFP expression at about 64 hours post-fertilization (hpf), in a few dispersed cells within a ventral-nasal region of the retina (Figure 2A, E), which at later stages were shown to differentiate into bipolar cells (Figure 3; see below). During the developmental period between 64 hpf and 72 hpf, the eGFP labelled cells increased in number and spread throughout a wider area, but the spatial patterns of the two transgenic lines were different. In the *xfz3 *line, eGFP expression is mainly restricted to the ventral half of the retina until 3 days post-fertilization (dpf) (Figure 2B, C), while eGFP-positive cells become more uniformly distributed in *xfz43 *individuals at an earlier stage (Figure 2F, G). By 5 dpf, both lines show higher densities of eGFP expressing cells that are uniformly distributed throughout the different quadrants of the retina (Figure 2D, H). This common temporal pattern parallels the onset of bipolar cell differentiation, which is known to occur at ~60 hpf [18]. The delayed detection of eGFP is probably reflecting the time required for production of Gal4-VP16 and subsequent activation of the UAS:eGFP cassette. Furthermore, the differences in eGFP expression between the two lines suggest that generation of different subsets of bipolar cells does not follow the same spatiotemporal pattern.

To validate whether reporter eGFP expression in the two lines was Gal4-VP16-dependent, we assessed the trans-activation by crossing with *UAS:RFP *transgenic fish (Methods). In both cases, the zebrafish larvae generated from these crosses showed co-expression of eGFP and RFP in retinal cells (Additional file 1: Figure S1), confirming that Gal4-VP16 proteins were present in these cells. However, trans-activation was not detected in the olfactory placodes of *xfz43;UAS:RFP *larvae (arrowheads), indicating a direct activation of eGFP in these structures. This finding is consistent with previous observations that transcription from the E1b minimal promoter in the UAS:eGFP cassette can in some cases be under the direct influence of a local enhancer [16].

Although, co-expression of eGFP and RFP was detected in retinal cells, we observed a higher number of RFP positive cells, particularly in *xfz43 *(Additional file 1: Figure S1). In addition, some of the eGFP positive cells did not show co-expression of RFP. These observations indicate that the expression of both these markers is variegated. Such variegation is frequently associated with Gal4 enhancer trapping in zebrafish [15,16]. Notably, the variegation implies that the spatiotemporal patterns of eGFP expression displayed by *xfz3 *and *xfz43 *(Figure 1 and Figure 2) are understatements of the Gal4-VP16 expression. Therefore, these eGFP patterns do not fully report on the enhancers that have been trapped.

### eGFP positive bipolar cells in xfz3 and xfz43 larvae display different morphologies

Direct identification of the eGFP-labelled retinal cells as bipolar cells was achieved by confocal microscopy analysis of retina tissue sections from 5 dpf larvae (Figure 3). For both transgenic lines eGFP expression was detected in cell bodies located in the outer part of the inner nuclear layer (INL), which correspond to the known location of bipolar cell bodies [18]. The central part of the retina contained higher densities of labelled cells. All eGFP positive cells also showed fluorescent labelling of their dendritic arbors in the outer plexiform layer (OPL), and their long axons extending into the inner plexiform layer (IPL). In addition, their axon terminals displayed specific patterns of foci within the sublaminae that are known to be characteristic of bipolar cells at early larval stages [19]. In *xfz3 *larvae, all the eGFP labelled cell bodies appeared to have similar locations in the outer part of the INL (Figure 3A, B), but their different patterns of foci within the IPL suggested that they represent two distinct morphological types of bipolar cells (Figure 3B, C). The eGFP positive cell bodies in the *xfz43 *line were more widely distributed in the INL (Figure 3D, E), and additional differences with respect to stratification within the IPL indicated the presence of more than one morphological type (Figure 3E, F).

### Distinct morphological types of bipolar cells located in the central retina of adults

In zebrafish, the different morphological types of bipolar cells have been classified based on adult retinae [7]. It is known that the axon terminals of bipolar cells undergo considerable morphological changes during early larval stages [19], and it was therefore important to also analyse the morphology of the eGFP positive bipolar cells in retinae from adult individuals.

**Figure 4:**
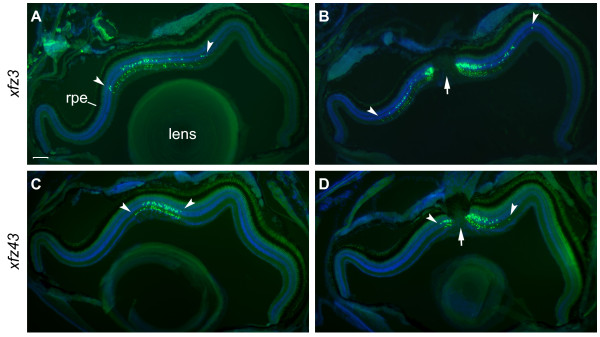
**Regional distribution patterns of eGFP labelled bipolar cells in adult retina**. Fluorescence images of retinal cross-sections from adult fish (6 months) from the transgenic lines *Tg(Gal4-VP16;UAS:eGFP)*^*xfz*3 ^(A, B), and *Tg(Gal4-VP16;UAS:eGFP)*^*xfz*43 ^(C, D). Sections were stained with an anti-GFP antibody (green) and counterstained with DAPI (blue). All images are oriented with the ventral side to the left. Arrowheads mark the central region containing eGFP labelled bipolar cells. The optic nerve is indicated by arrows (B, D). Scale bar: 100 μm. Abbreviation: rpe, retinal pigmented epithelium.

Our analysis of the overall distribution of eGFP expressing bipolar cells by fluorescence microscopy of serial cross-sections of adult retina revealed highly regionalized patterns in both transgenic lines (Figure 4). The eGFP labelled cells in *xfz3 *were restricted to a central region with a radius corresponding to about one third of the whole retina (Figure 4A, B). Within this central region of the retina, we observed a significantly higher density of labelled bipolar cells surrounding the optic nerve, with the majority located on the dorsal side of it (Figure 4B). Similarly, adult *xfz43 *fish showed a high density of eGFP positive cells near the optic nerve, which were mainly concentrated in a narrow region on the dorsal side (Figure 4D). However, in this line we did not detect a wider distribution of more dispersed fluorescent cells (Figure 4C, D). Our analysis of retina from *xfz43 *larvae at 10 and 12 dpf (Additional file 3: Figure S3L, Q; see below), indicated that regional restriction of the labelled bipolar cells was initiated at these larval stages.

In the two enhancer trap lines, the eGFP labelling of the bipolar cells delineates the cell bodies, dendrites, axons and their terminals. Importantly, the layers of axon terminal ramification within the IPL and the size of the terminals have been used as markers to identify 17 different morphological types of bipolar cells in zebrafish [7]. In order to compare the bipolar cells labelled in *xfz3 *and *xfz43 *with these known morphological types, serial sections of adult retina were used as material for detailed analysis by confocal microscopy.

**Figure 5:**
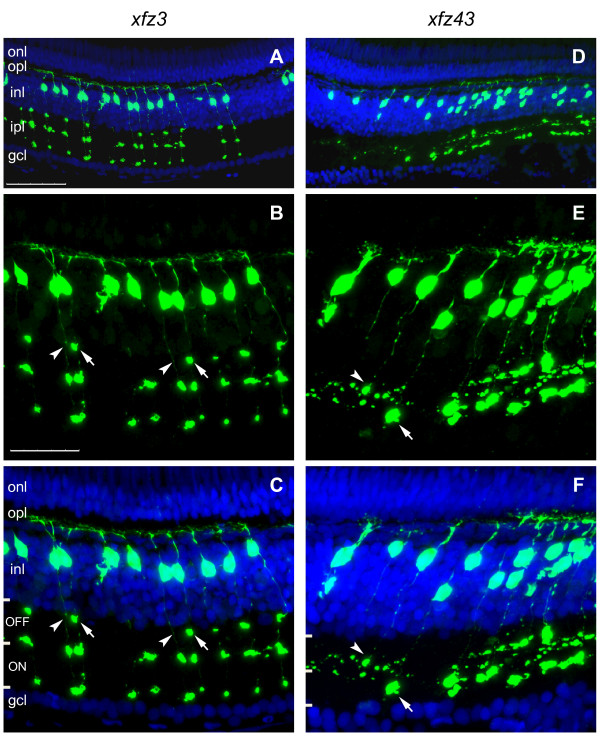
**Axon terminal patterns within the adult retina define distinct types of bipolar cells**. (A-C) Confocal images of a retinal cryosection, labelled with an anti-GFP antibody (green) and counterstained with DAPI (blue), from an adult *Tg(Gal4-VP16;UAS:eGFP)*^*xfz*3 ^fish (6 months). Higher magnification images of the middle part in (A) are shown in (B, C). The image in (B) is the same as in (C) but without DAPI. Arrows in (B, C) indicate a bouton-like axon terminal structure in the OFF layer, which is present in one bipolar cell type but absent in the other (arrowheads). (D-F) Retinal cryosection from an adult *Tg(Gal4-VP16;UAS:eGFP)*^*xfz*43 ^fish. The confocal images are arranged in the same way as shown for the other line. Arrows in (E, F) indicate a large bouton-like structure in the ON sublamina, which is present at the axon terminal of one bipolar cell type. A second type of bipolar cells shows terminal arborisation within the OFF sublamina (arrowheads). ON and OFF sublamina of the inner plexiform layer are indicated by bars in (C, F). Scale bars: 50 μm (A, D); 25 μm (B, C, E, F). Abbreviations: gcl, ganglion cell layer; inl, inner nuclear layer; ipl, inner plexiform layer; onl, outer nuclear layer; opl, outer plexiform layer.

The somata of all the eGFP positive bipolar cells in the retina of *xfz3 *fish are located in similar distal positions within the INL (Figure 5A–C). However, they display clear differences within the IPL, where the members of one group have three bouton-like structures, whereas the other bipolar cells characteristically have two of these axon terminal structures (Figure 5B, C). On the basis of these features and the positions of the bouton-like structures within specific sublayers of the ON and OFF sublaminae, the two subsets of *xfz3 *bipolar cells appear to correspond to the previously described B_**OFF**_-s1/s3/s5/6 and B_**ON**_-s3/s5 types, respectively [7,20].

In the *xfz43 *line, the labelled bipolar cell bodies are located both at distal and middle positions within the INL, and these two subsets are also different with respect to their axon terminals (Figure 5D–F). The members of the distal group show arborisation of their terminals within the OFF sublamina and can be classified as a B_**OFF**_-s2/s3 type [7]. By contrast, each of the bipolar cells located in the middle of the INL only have a single, large terminal bouton within the ON sublamina, indicating that they correspond to the previously described B_**ON**_-s4 type [7].

**Figure 6:**
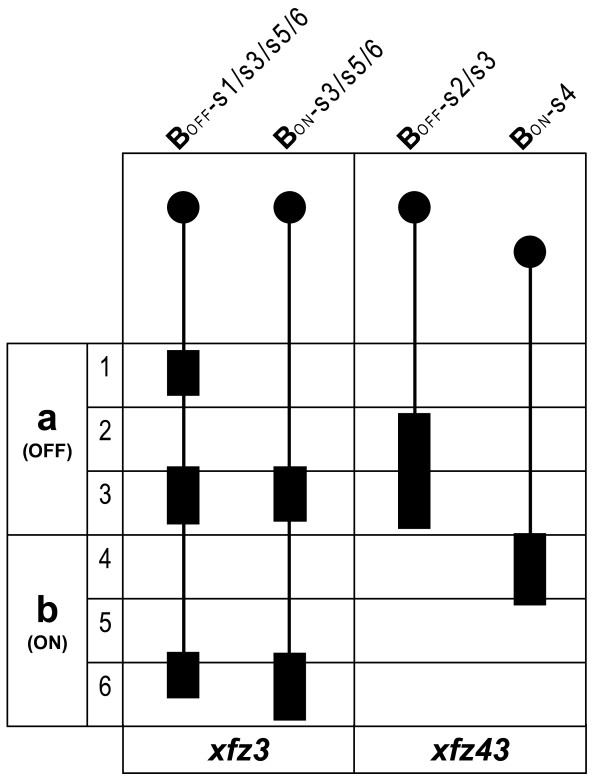
**Schematic illustration of the bipolar cell types identified in the enhancer trap lines**. The sublayers (s1–6) within sublamina **a **(OFF) and **b **(ON) of the IPL are indicated, and the basal-apical orientation is shown with basal at the bottom. Cell bodies are shown as filled circles and their relative positions within the INL are indicated. The axon terminal structures are shown as filled boxes.

**Figure 7:**
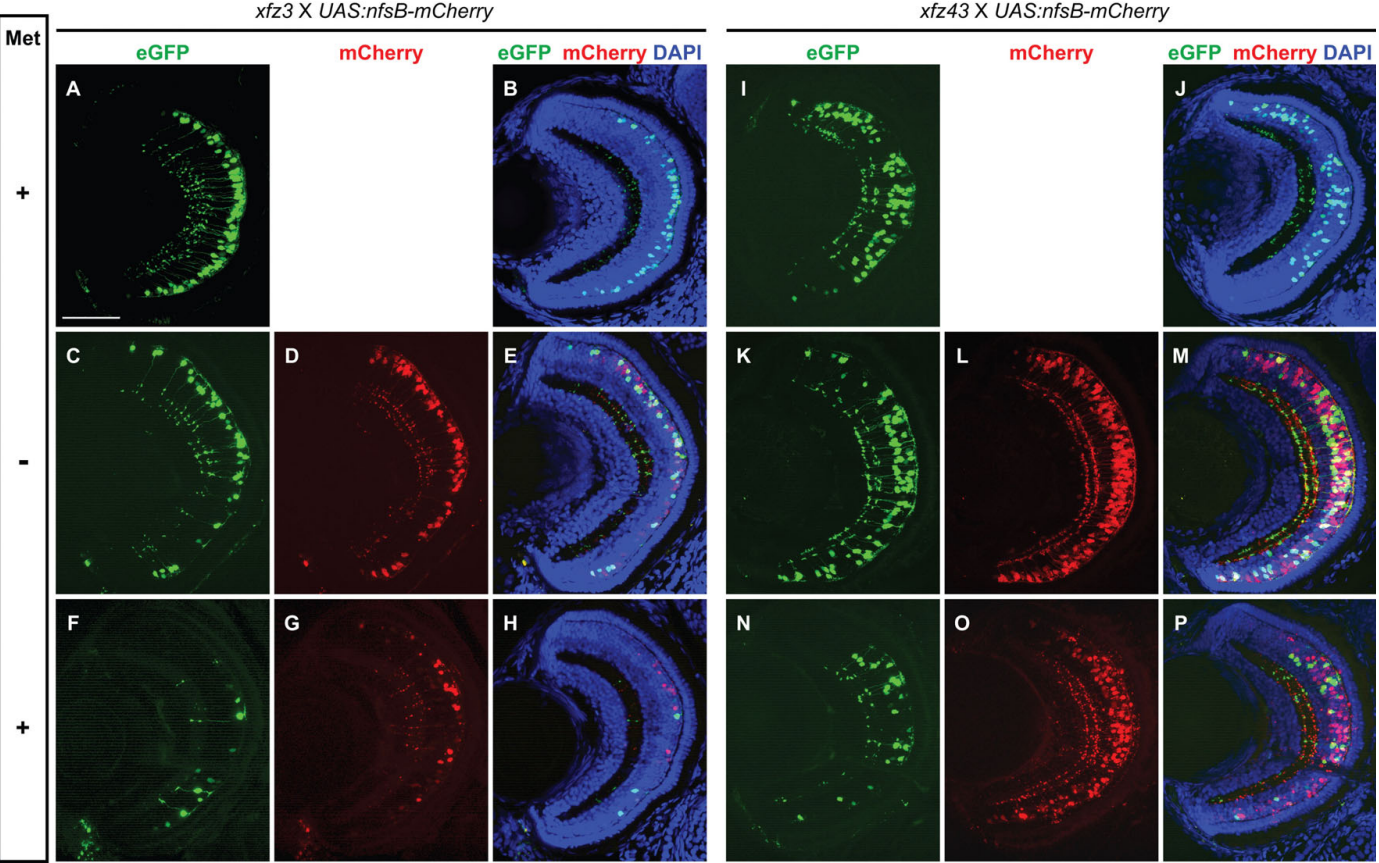
**Prodrug dependent ablation of specific bipolar cell types in larvae from enhancer trap lines**. (A-H) Confocal images of retinal cross-sections from larvae (5 dpf) produced by mating of *UAS:nfsB-mCherry *transgenic fish to *Tg(Gal4-VP16;UAS:eGFP)*^*xfz*3^. (A, B) Retina from a larva without the *UAS:nfsB-mCherry *fusion gene that was unaffected by Met treatment. (C-E) Retina from larva expressing NTR-mCherry (red) in bipolar cells. (F-H) Retina from larva expressing NTR-mCherry (red) showed loss of fluorescently labelled bipolar cells following Met treatment. (I-P) The same type of analysis as in (A-H) conducted on larvae produced by mating of *UAS:nfsB-mCherry *transgenic fish to *Tg(Gal4-VP16;UAS:eGFP)*^*xfz*43^. (N-P) Significant degeneration and loss of labelled bipolar cells occurred in the retina of Met treated larvae. Labelling to the left indicate Met treatment (+) and no treatment (-). The crosses and different types of fluorescence are indicated at the top. Scale bar: 50 μm.

Our analysis shows that the distinct features of these four different bipolar cell types closely match the descriptions of known morphological types (Figure 6; [7,20]). However, we cannot exclude the possibility that one or more of the eGFP labelled cells may represent previously uncharacterized bipolar cell types.

### Targeted ablation and recovery of specific bipolar cell types

It has been demonstrated previously that Gal4-VP16/eGFP enhancer trap lines can be utilized to induce tissue-specific cell death by breeding to *Tg(UAS:nfsB-mCherry) *fish, which produce a fusion protein consisting of *E. coli *nitroreductase B (NTR) and the fluorescent marker mCherry [16]. The prodrug metronidazole (Met) is converted into a cytotoxic DNA cross-linking agent in cells expressing the fluorescent NTR-mCherry fusion protein, leading to specific ablation of the labelled cells by apoptosis [16,21,22].

To investigate whether this NTR/Met system could be used to achieve specific ablation of bipolar cells, adults from the *xfz3 *and *xfz43 *lines were mated to *Tg(UAS:nfsB-mCherry) *fish to obtain compound heterozygous larvae displaying both eGFP and mCherry fluorescence. Notably, it was revealed by confocal microscopy of retina cross-sections that expression of eGFP and NTR-mCherry in each of these larvae occurred both within the same cells and separately in different subpopulations of the same types of bipolar cells (Figure 7C–E and 7K–M). These features are likely to reflect variegated expression of both markers. However, the larvae from both crosses showed significantly higher numbers of cells with mCherry labelling, indicating that activation of the UAS:eGFP cassette by Gal4-VP16 is less efficient than for UAS:nfsB-mCherry.

Larvae obtained from crosses between *Tg(UAS:nfsB-mCherry) *fish and the two enhancer trap lines were treated for 24 h with Met from 4 dpf till 5 dpf (Methods). For both types of mating, comparisons of retina cross-sections from treated and untreated individuals at 5 dpf revealed significant differences with respect to the numbers and shapes of the fluorescently labelled cells (Figure 7). In the case of larvae generated from the *xfz3 *mating, the pattern of fluorescently labelled axon terminals within the IPL was disrupted and most of the eGFP and mCherry positive cells were ablated by the 24 h prodrug treatment (Figure 7F–H). In addition, the few remaining mCherry labelled cell bodies were rounded and/or reduced in size, and they generally lacked axons, suggesting that they were undergoing apotosis as has been reported for the effects of the NTR/Met system on other cell types [21-23].

**Figure 8:**
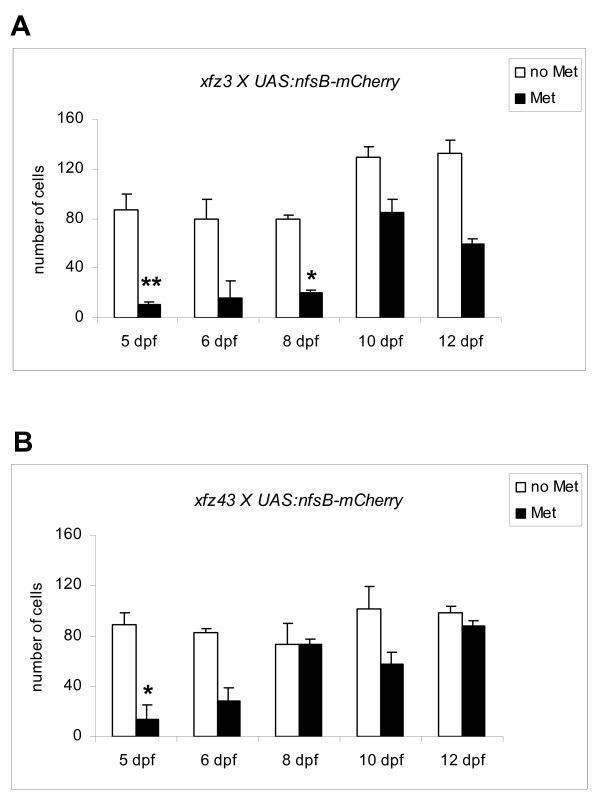
**Quantitative analysis of eGFP labelled bipolar cells during recovery from prodrug treatment**. The diagrams show comparisons of numbers of eGFP positive bipolar cells in Met treated and untreated siblings, following removal of the prodrug at 5 dpf. (A) Comparison of bipolar cell numbers in Met treated larvae from the *xfz3 *× *UAS:nfsB-mCherry *mating with their untreated siblings. (B) Comparison of bipolar cell numbers in Met treated larvae from the *xfz43 *× *UAS:nfsB-mCherry *mating with their untreated siblings. Cell numbers were obtained by counting of all the eGFP positive cells located within 30 μm stacks from cross sections through the central part of the retina. Columns represent average values from 2–4 cross sections. (*p < 0.05, **p < 0.01, Student's *t*-test). Error bars, s.d.

Quantitative analysis showed more than 85% loss of eGFP labelled bipolar cells in Met treated larvae from the *xfz3 *mating (Figure 8A), indicating that most of these cells were co-expressing the NTR-mCherry fusion protein. One notable exception was a few eGFP positive cells that displayed irregular/immature axons and terminals (Figure 7F). Possibly, these cells may be immature bipolar cells that started differentiation during the prodrug treatment. Alternatively, they may have survived due to lack of (or very low) expression of NTR-mCherry fusion protein.

Analysis of the retina of *xfz43*; *Tg(UAS:nfsB-mCherry) *larvae immediately after completing the prodrug treatment also showed nearly 85% loss of both eGFP and mCherry labelled bipolar cells (Figure 7N, O and Figure 8B). Ablation of the majority of the eGFP positive cells was probably a consequence of their co-expression of the NTR-mCherry fusion protein. The few remaining eGFP labelled cells may be explained in the same ways as suggested for *xfz3*; *Tg(UAS:nfsB-mCherry) *larvae (see above). We also observed a significant removal of the bipolar cells that exclusively expressed the NTR-mCherry fusion protein (Figure 7L, O). However, for many of these mCherry positive cells, small-sized cell bodies and axon terminals with irregular features were still detectable at 5 dpf, indicating ongoing apoptosis (Additional file 4: Figure S4). In support of this interpretation, we observed more complete removal at 6 dpf and 8 dpf (Additional file 3: Figure S3D, I).

To investigate whether the retina of larvae with ablated bipolar cells were able to recover, we quantified the eGFP positive cells, which were detected by confocal microscopy (Additional file 2: Figure S2 and Additional file 3: Figure S3), during an additional period of seven days, until 12 dpf (Figure 8). The first three days after termination of prodrug treatment (until 8 dpf), *xfz3*; *Tg(UAS:nfsB-mCherry) *larvae showed only a small increase in the number of eGFP labelled bipolar cells (Figure 8A). By 10 dpf, we observed a significantly higher number of these cells, which was comparable to their number at 8 dpf in the untreated siblings expressing only Gal4-VP16/eGFP (Figure 8A). However, a clear increase in the population of eGFP positive bipolar cells also occurred between 8 dpf and 10 dpf in the untreated *xfz3 *siblings (Figure 8A). This suggests that the partial recovery of eGFP labelled neurons observed during the 8–10 dpf period mainly reflects a normal increase in the production of these particular bipolar cell types.

The reduced number of eGFP positive cells at 12 dpf as compared to 10 dpf probably does not reflect a loss of these labelled bipolar cells. If labelled cells were lost during this period, it is likely that it would involve apoptosis. However, apoptotic cells were not detected at these later stages. Therefore, we assume that the fluctuation is mainly due to variations in cell numbers between different individuals. The estimates of eGFP positive cells in sections from untreated siblings also showed a fluctuation that may be explained in this way. Similar considerations may apply to our estimates for *xfz43 *(Figure 8B; see below).

Quantification of eGFP positive cells following Met treatment of larva from the *xfz43 *mating showed a similar degree of elimination, but the subsequent recovery of labelled bipolar cells seemed to occur more rapidly and their number was almost completely restored as compared to the untreated siblings (Figure 8B and Additional file 3: Figure S3). Although we noted some fluctuations in our estimates of eGFP positive cells, the ablation experiment seemed to induce a process of recovery involving active regeneration of the two types of labelled bipolar cells in the *xfz43 *line. This interpretation is also supported by observations of differences between Met treated *xfz43; Tg(UAS:nfsB-mCherry) *larvae and their untreated siblings with respect to retinal distribution of labelled bipolar cells at later stages. In the retina of untreated *xfz43 *larvae, labelled bipolar cells showed regional restrictions at the dorsal and ventral margins (Additional file 3: Figure S3L, Q), indicating that the two types of labelled bipolar cells are no longer generated in the peripheral regions at these stages. By contrast, the generation of new fluorescently labelled bipolar cells in the retina of prodrug treated individuals did not show such regional restrictions (Additional file 3: Figure S3R-T). This is likely to reflect an induced production of labelled bipolar cells in the entire retina, including the otherwise inactive peripheral parts.

The targeted ablation did not seem to affect retinal lamination at any of the stages analysed (Additional file 2: Figure S2 and Additional file 3: Figure S3). We also observed that the newly generated bipolar cells in Met treated larvae from both transgenic lines had re-established quite normal patterns of axon terminals within the IPL at 12 dpf (Additional file 2: Figure S2 and Additional file 3: Figure S3). Furthermore, we detected large aggregates of fluorescent debris from ablated cells, particularly in *xfz43; Tg(UAS:nfsB-mCherry) *individuals, which were still present in the eyes at later stages of recovery (Additional file 3: Figure S3N, S). Notably, Met treatment did not seem to affect the overall health and survival of the larvae, and some of these were raised to adulthood (data not shown).

## Discussion

The realization that the different classes of retinal neurons consist of multiple morphologically distinct cell types represents an extra challenge to the research aimed at understanding the development, organization, and function of the vertebrate retina. In relation to this issue, we have generated enhancer trap lines of zebrafish in which specific types of bipolar cells are co-expressing a fluorescent marker protein (eGFP) and a modified transcription activator (Gal4-VP16), providing highly specific tools for *in vivo *visualization of these cells and perturbation of their differentiated state and function. Our studies of these lines revealed novel regional patterns of bipolar cell distribution, and demonstrated how they can be applied to induce and monitor targeted ablation and how the retina recovers by generation of new neurons.

### Genetic labelling and visualization of distinct bipolar cell types in zebrafish larvae

Previous attempts to establish transgenic lines of zebrafish in which fluorescent marker proteins are expressed specifically by a subset of retinal interneurons have utilized known promoters [19,24,25]. Here we describe two transgenic lines, *xfz3 *and *xfz43*, generated by enhancer trapping that show specific expression of the fluorescent marker eGFP in distinct types of bipolar cells. This was achieved by using a previously developed transposon vector containing a hybrid transcription activator Gal4-VP16 and a UAS:eGFP reporter cassette [16]. Hence both enhancer trap lines co-expressed Gal4-VP16 and eGFP in particular subsets of bipolar cells. Importantly, these interneurons also showed strong eGFP labelling of axons and dendrites, facilitating identification of specific morphological types. Our analysis of the retina from larvae by confocal microscopy indicated the presence of two distinct types of labelled bipolar cells in each line.

Both transgenic lines display temporal expression patterns correlating well with the onset of bipolar cell differentiation in the ventral-nasal region of the retina, previously known to occur at ~60 hpf [18]. However, the two lines show some divergence in their spatial patterns at later stages when maturation of the bipolar cells spread to other parts of the retina. If the expression of eGFP directly reflects differentiation of these bipolar cells, this would suggest responses to different combinations of inductive signals distributed within the developing retina. This idea also seems to be consistent with the previous reports that the topographical spread of differentiating rods and double cones show irregular patterns [18,26]. Hence, it is unclear how the generation of photoreceptors and specific bipolar cell types may relate to the simple wave-like spread of differentiating retinal ganglion cells and amacrine cells from the ventral-nasal region, which is known to depend on a wave of Sonic hedgehog expression in cooperation with other factors [27,28].

Identification of the endogenous genes that presumably are regulated by the enhancers controlling Gal4-VP16/eGFP expression in the transgenic lines may provide new clues regarding the mechanisms responsible for the differentiation of specific types of bipolar cells. However, our analysis of the candidate genes located within regions of ~50 kb (*xfz3*) and ~400 kb (*xfz43*) relative to the vector insertion sites on chromosome 2 and 6, respectively, did not identify any gene with correlating retinal expression. Although further work will be required to identify these genes, whose enhancer elements may be located more than a megabase away [12,29,30], the activity of Gal4-VP16 can be utilized to induce ectopic expression of other genes suspected to play roles in bipolar cell differentiation. This implies that the two enhancer trap lines will be valuable tools in future investigations of how the distinct morphological types of bipolar cells are generated and what their functional significance may be.

### Bipolar cell types with regionalized distribution and distinct axon terminal stratification patterns in the adult retina

The retinal pattern of ganglion cells is known to correspond with the retinotectal map, and photoreceptor types are in many cases distributed asymmetrically across the surface of the retina [31-33]. However, it is still unclear whether such regionalized distribution is a common feature of bipolar cells as well [31]. Notably, our analysis of adult retina from the enhancer trap lines showed highly regionalized distribution of the different types of eGFP expressing bipolar cells. In both lines the labelled cells were restricted to the central region of the retina surrounding the optic nerve, but the patterns of distribution and degree of restriction were clearly different. We also noted that eGFP labelled cells disappeared from the dorsal and ventral parts of the retina at later larval stages of these transgenic lines, indicating a progressive regional restriction from larva to adult. These observations suggest some kind of functional specialization of these particular types of bipolar cells and may reflect a similar regional distribution of their synaptic partners among the photoreceptors.

Previous studies of bipolar cells in the retina of adult zebrafish classified 17 distinct morphological types mainly on the basis of characteristic features of the axon terminals within the IPL [7]. Strong fluorescent labelling of the axons of the eGFP expressing bipolar cells in the two transgenic lines facilitated comparison with the known morphological types. This analysis revealed that the two subsets of labelled bipolar cells in the *xfz3 *line most closely resemble the previously described B_**OFF**_-s1/s3/s5/6 and B_**ON**_-s3/s5 types [7,20]. For the eGFP positive bipolar cells in the second line (*xfz43*), we also found good correspondence to two morphological types (B_**OFF**_-s2/s3 and B_**ON**_-s4). Although it remains to verify these morphological types, it seems clear that the subset of labelled bipolar cells in both enhancer trap lines include ON and OFF types. Previously, the upstream regulatory sequence from the *nyx *gene has been used in combination with the Gal4-UAS system to generate transgenic zebrafish with expression of membrane targeted yellow fluorescent protein (MYFP) in multiple types of ON-bipolar cells [19]. Detailed analysis of this transgenic line was only performed on larval stages, and comparisons with the morphological classification in adults precluded direct identification of specific types of bipolar cells.

### Transgene and prodrug-dependent ablation of specific bipolar cell types

The Gal4-UAS system has been used most extensively in *Drosophila *to activate many different transgenes in the same cells that express Gal4 [34]. Similarly, the zebrafish enhancer trap lines described in this report may be applied as tools to study the differentiation and physiology of the specific types of Gal4/eGFP expressing bipolar cells. In particular, these transgenic lines may be used to study how distinct types of bipolar cells differentiate and generate specific patterns of axon terminals within the IPL during larval development [19]. Their potential as tools for investigating these processes was established from our observation of eGFP expression at the onset of bipolar cell differentiation and our demonstration that the Gal4-UAS system could be applied to drive early transgene expression of RFP in these cells.

The zebrafish retina has the ability to regenerate [35,36], and this research could also benefit from applications of the Gal4-UAS system that have been shown to facilitate targeted cell ablation [16,21,22]. To test whether the bipolar cell expressing enhancer trap lines can be used for this purpose, they were mated to *UAS:nfsB-mCherry *transgenic fish to produce larvae with Gal4-VP16 induced retinal expression of a fluorescent fusion protein, nitroreductase-mCherry (NTR-mCherry). It was confirmed that this fusion protein was co-expressed with Gal4-VP16 and eGFP in specific bipolar cells, and targeted ablation of these cells occurred when larvae were treated with the prodrug Metronidazole (Met).

We used confocal microscopy to analyse in detail the effects of Met treatment and to monitor how the retina recovered after removal of the prodrug. In accordance with previous studies of the effects of the cytotoxic agent produced by the NTR/Met system [21-23], the targeted bipolar cells were rounded and reduced in size indicating ongoing apoptosis. For both transgenic lines we observed that mCherry labelled bipolar cells were almost completely ablated. Despite this extensive cell death, which also generated large aggregates of cell debris, the general architecture of the neural retina seemed unaffected.

Following removal of the prodrug, the transgenic lines quickly generated new fluorescently labelled bipolar cells, and recovery was almost complete after seven days. Although an increase in the number of labelled bipolar cells was also observed in untreated larvae during the same period, our quantitative analysis strongly suggests that the recovery, particularly in one of the transgenic lines (*xfz43*), was mainly due to induction of an active regeneration process. These results suggest that some aspects of retina regeneration can be further investigated in larvae from one (*xfz43*) or both transgenic lines described in this report. As the NTR/Met system was recently shown to work well in adult zebrafish [37], it may also be possible to use the transgenic line(s) to study retina regeneration in adults.

## Conclusion

This work applies transgenic techniques, which have recently been established in zebrafish, to label and manipulate specific subsets of cells belonging to a particular type of retinal neurons, the bipolar cells. Previous research has shown that the bipolar cells can be classified into 17 different morphological types, mainly on the basis of differences in their axon terminal ramifications within the IPL. Further investigations of the properties of the various subtypes of bipolar cells will require specific molecular markers and genetic techniques that can facilitate analyses *in vivo*. We describe the generation of two enhancer trap lines in which specific subsets of identifiable types of bipolar cells show co-expression of a fluorescent marker (eGFP) and a transcription activator (Gal4-VP16). This labelling revealed a regionally restricted distribution of bipolar cell subtypes that was previously unknown and may indicate regional specializations within the retina. To demonstrate the utility of the two transgenic lines, we used the Gal4-UAS system to drive expression of a bacterial nitroreductase fusion protein (NTR-mCherry) in the labelled bipolar cells of zebrafish larvae, which in combination with the prodrug metronidazole caused efficient ablation of these cells without affecting the retinal architecture. This experiment also provided some evidence that the larval retina can compensate the loss of bipolar cells by active regeneration. Hence, the enhancer trap lines described in this study will provide valuable tools for further investigations of the generation and function of specific types of bipolar cells.

## Methods

### Zebrafish

Zebrafish and embryos were maintained and bred as described elsewhere [38]. All embryos were obtained from natural mating, and pigmentation was prevented by adding 0.003% phenylthiourea (PTU, Sigma) to the E3 medium. Two Gal4-VP16 enhancer transgenic lines, *Tg(Gal4-VP16, UAS:eGFP)*^*xfz*3 ^and *Tg(Gal4-VP16, UAS:eGFP)*^*xfz*43^, which are also referred to as *xfz3 *and *xfz43*, respectively, were generated as described previously [16], and identified from a small scale screen in our laboratory [17]. The insertions were mapped by LM-PCR as described in [16], and Blast searches were performed at the ENSEMBL genome website (zebrafish assembly version 7, Zv7). We identified the *xfz3 *insertion at position 528.484 on chromosome 2, and the *xfz43 *insertion at position 32.830.217 on chromosome 6 (Additional file 5: Table S1). Four candidate genes, which were located close to the insertion site in each line, were analysed by whole mount in situ hybridisation to compare expression patterns. The four *xfz3 *candidate genes were: ENSDART00000040838, zgc:113518, ENSDARESTG00000013190, and ENSDARESTG00000013221. The genes surrounding the *xfz43 *insertion were: ENSDART00000084419, ENSDARESTG00000007490, ENSDARESTG00000007489, and tardbp.

The two lines will be deposited in The Zebrafish International Resource Center (ZIRC). The *UAS:RFP *transgenic fish harbouring the plasmid T2ZUASRFP [14] was identified and used as in [17]. To examine if the *xfz3 *and *xfz43 *transgenic lines drive expression in *trans *through a UAS element, we crossed *xfz3 *and *xfz43 *to the *UAS:RFP *transgenic line. Double transgenic embryos were analyzed for eGFP and RFP by using a fluorescence stereoscope (Zeiss SteREO Lumar, Zeiss). The transgenic fish harbouring nitroreductase B, *Tg(UAS-E1b:NfsB-mCherry)*^*c*264 ^[16], was purchased from ZIRC.

### Ablation and regeneration

*Tg(UAS-E1b:NfsB-mCherry)*^*c*264 ^was crossed with *xfz3 *or *xfz43*. Heterozygous larvae that express both eGFP and mCherry were incubated with 10 mM of the prodrug metronidazole (Met, Sigma) for 24 hours, from 4 to 5 days post-fertilization (dpf). The Met solution was washed away with at least three changes of fresh E3 medium, as also described previously [37], and larvae were fed as wild type fish and sampled at selected time points post treatment.

### Cryosectioning

Larvae and adult eyes were fixed in 4% paraformaldehyde, cryoprotected in 30% sucrose, subsequently embedded in OCT compound Medium, and sectioned at 30 μm thickness. Sections were dried at 50°C for 2 hours, and stored at -20°C.

### Immunohistochemistry on sections

Tissue sections were re-hydrated with PBS, and immunohistochemistry was performed as described previously [39]. The polyclonal rabbit anti-GFP primary antibody (Torrey Pines Biolabs) was used at a 1:500 dilution, while the secondary antibody, anti-rabbit Alexa 488 (Molecular Probes), was used at a 1:200 dilution. Nuclei were visualized with 1 mg/L solution of DAPI (Molecular Probes). Slides were coverslipped with anti-fade mounting media [39]. In the ablation and regeneration experiments, sections were stained with DAPI directly after sectioning and re-hydration, and direct fluorescence from eGFP was visualized without use of immunohistochemistry.

### Imaging

Fluorescent images were captured by either Zeiss SteREO Lumar (Zeiss) or Leica TCS SP5 (Leica). Z-stack images were obtained at 0.5 μm intervals, and compacted into one image using Leica Application confocal software. Figures were generated using Adobe CS2 Photoshop and Illustrator. Image contrast was enhanced to one individual image at a time.

## Abbreviations

dpf: days post fertilization; GCL: ganglion cell layer; GFP: green fluorescent protein; INL: inner nuclear layer; IPL: inner plexiform layer; Met: metronidazole; NTR and nfsB: nitroreductase B; ONL: outer nuclear layer; OPL: outer plexiform layer; RPCs: retinal progenitor cells; UAS: upstream activator sequences.

## Authors' contributions

XFZ carried out all the experiments, prepared the figures, participated in design and helped to draft the manuscript. SE conceived the study, designed the experiments, supervised its progress and helped to draft the manuscript. AF conceived the study, designed the experiments, supervised its progress and wrote the manuscript. All authors read and approved the final version of the manuscript.

## Supplementary Material

Additional file 1 **Figure S1. Transactivation of a UAS-regulated gene in retinal bipolar cells of the enhancer trap lines**. Induced expression of red fluorescent protein (RFP) from a UAS:RFP construct in larvae at 3 dpf demonstrated for both transgenic lines the presence of Gal4-V16 in retinal bipolar cells, and its absence from the olfactory placodes in *xfz43 *(arrowheads). Scale bar: 50 μm. Click here for file

Additional file 2 **Figure S2. Retina recovery in larval progeny from the *xfz3 *× *UAS:nfsB-mCherry *mating**. Retinal cryosections were obtained from larvae at different time points during a period of seven days following removal of Met at 5 dpf. The left panel (labelled 'no Met' at the top) show confocal images of retina cross-sections from untreated siblings expressing only Gal4-VP16/eGFP. The right panel (labelled 'Met 4–5 dpf') show confocal images of retina cross-sections from NTR-mCherry expressing larvae following removal of Met. The different time points and corresponding larval stages are: (C-E) 1 day post-treatment (6 dpf); (H-J) 3 days (8 dpf); (M-O) 5 days (10 dpf); (R-T) 7 days (12 dpf). The different types of fluorescence are indicated at the top. Scale bar: 50 μm. Click here for file

Additional file 3 **Figure S3. Retina recovery in larval progeny from the *xfz43 *× *UAS:nfsB-mCherry *mating**. Confocal images of retina cross-sections from different stages of untreated siblings, which express only Gal4-VP16/eGFP (left panel), and Met treated larvae expressing NTR-mCherry (right panel) are arranged in the same way as described in Additional file 2: Figure S2. Scale bar: 50 μm. Click here for file

Additional file 4 **Figure S4. Apoptotic cells in retina of prodrug treated larvae from the *xfz43 *× *UAS:nfsB-mCherry *mating**. (A, B) Higher magnifications are shown for areas from Figure 7L, O, respectively. Retina from Met treated larva (B) show rounded NTR-mCherry labelled cells lacking axons. The axon termini in both the IPL and OPL (arrowheads) are also disintegrating. Scale bar: 25 μm. Click here for file

Additional file 5 **Table S1. Genomic sequences flanking vector insertions**. Zebrafish genomic sequences flanking the Tol2 vector insertions associated with retinal expression of Gal4-VP16/eGFP in the two transgenic lines. Tol2 vector sequences are underlined and coloured (right arm in red and left arm in blue). (Format: DOC). Click here for file
