# Supplementary figures and images for: Labelling and targeted ablation of specific bipolar cell types in the zebrafish retina
Source: BMC Neurosci. 2009 Aug 27;10:107. doi: 10.1186/1471-2202-10-107 (PMC3224687; doi:10.1186/1471-2202-10-107)

*xfz3* x *UAS:RFP*

*xfz43* x *UAS:RFP*

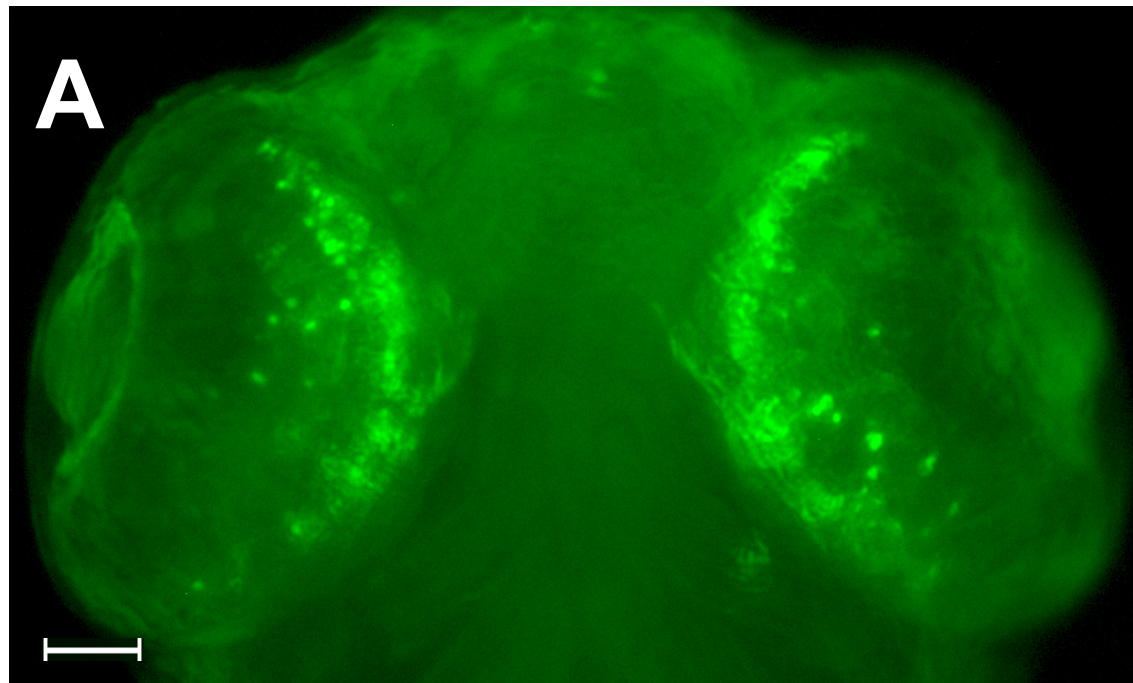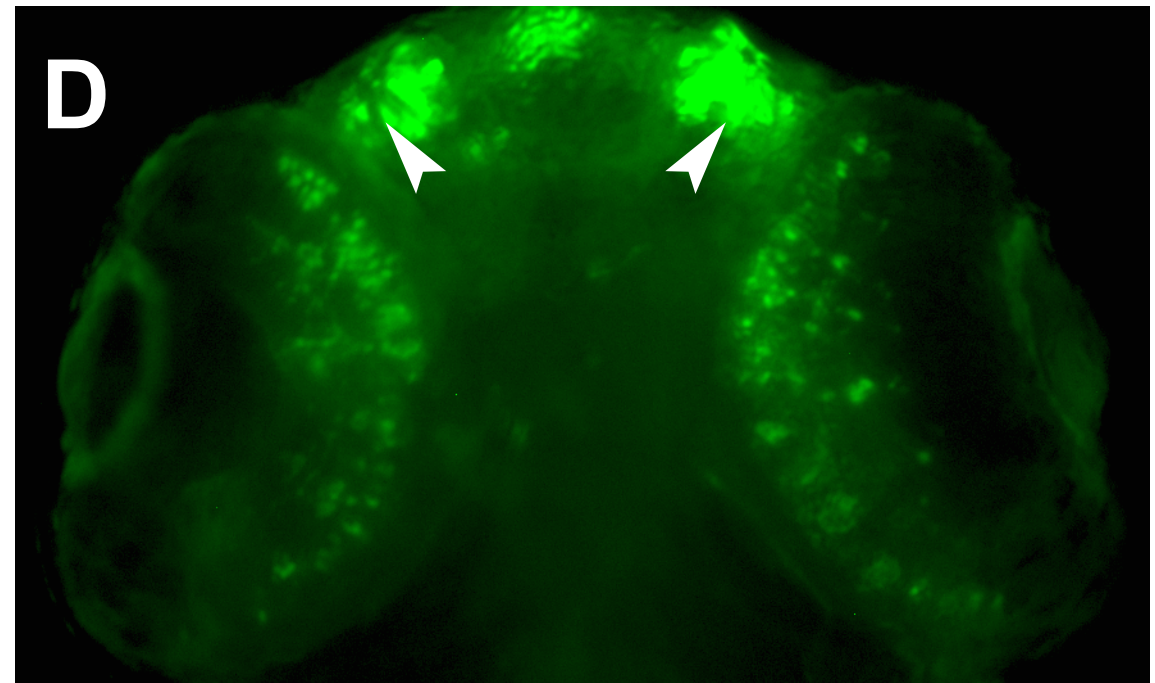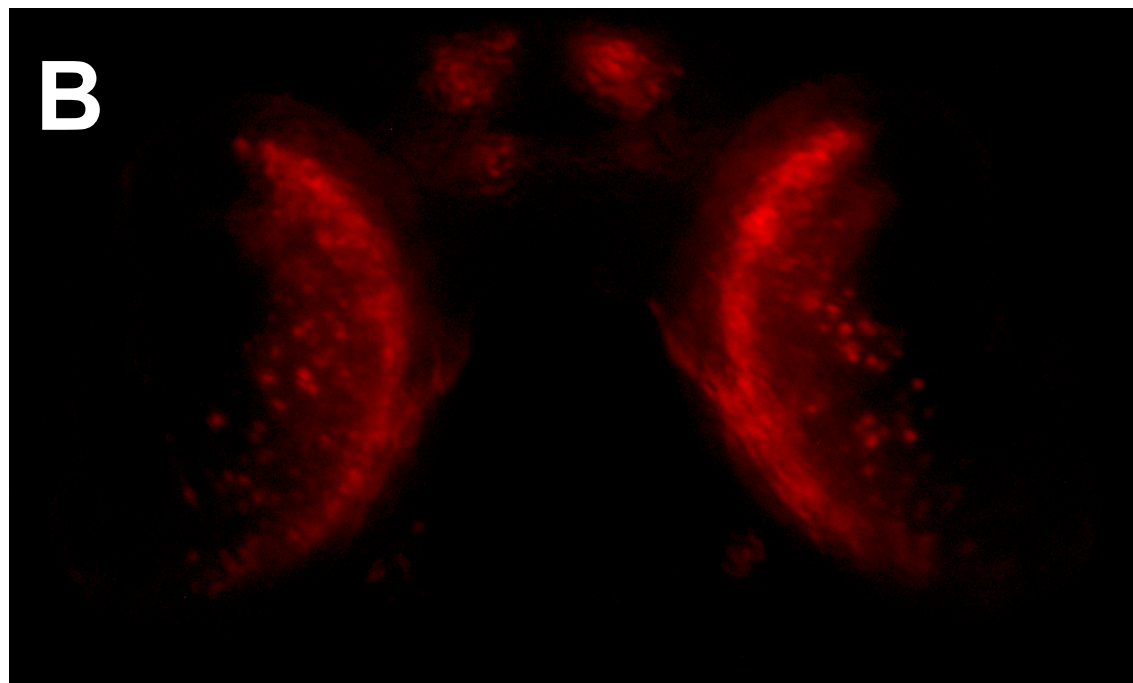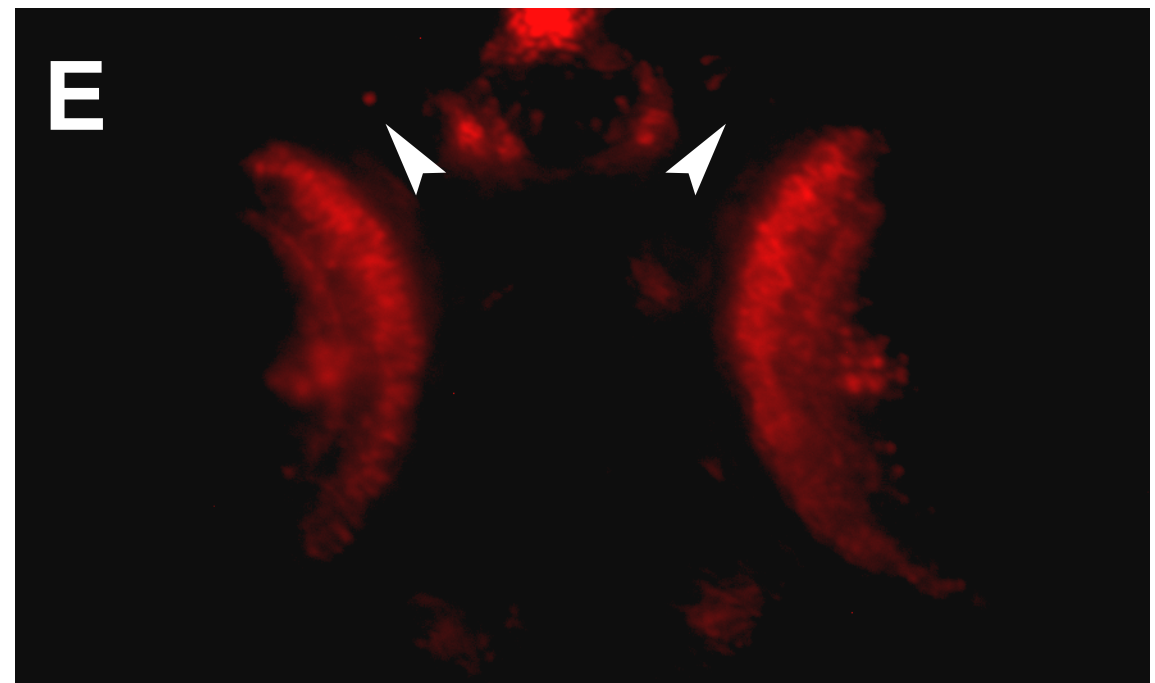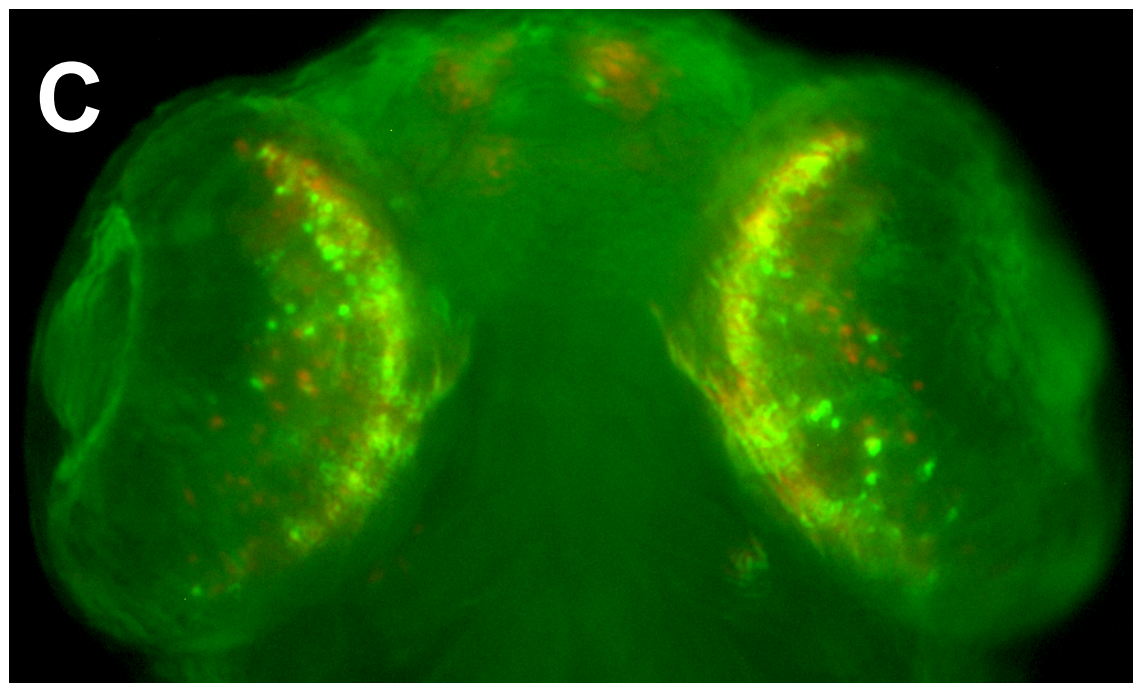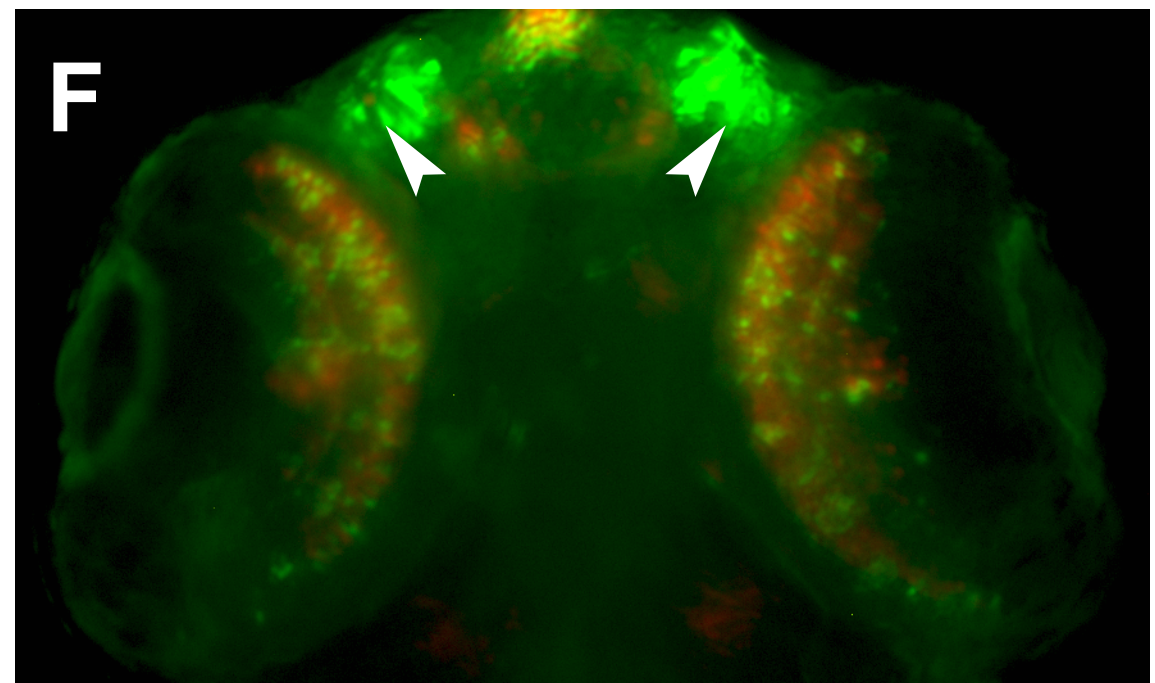

GFP

RFP

Merge

Supplement: Additional file 1 — Figure S1. Transactivation of a UAS-regulated gene in retinal bipolar cells of the enhancer trap lines. Induced expression of red fluorescent protein (RFP) from a UAS:RFP construct in larvae at 3 dpf demonstrated for both transgenic lines the presence of Gal4-V16 in retinal bipolar cells, and its absence from the olfactory placodes in xfz43 (arrowheads). Scale bar: 50 μm. [file 1471-2202-10-107-S1.pdf]

*xfz3* X *UAS:nfsB-mCherry*

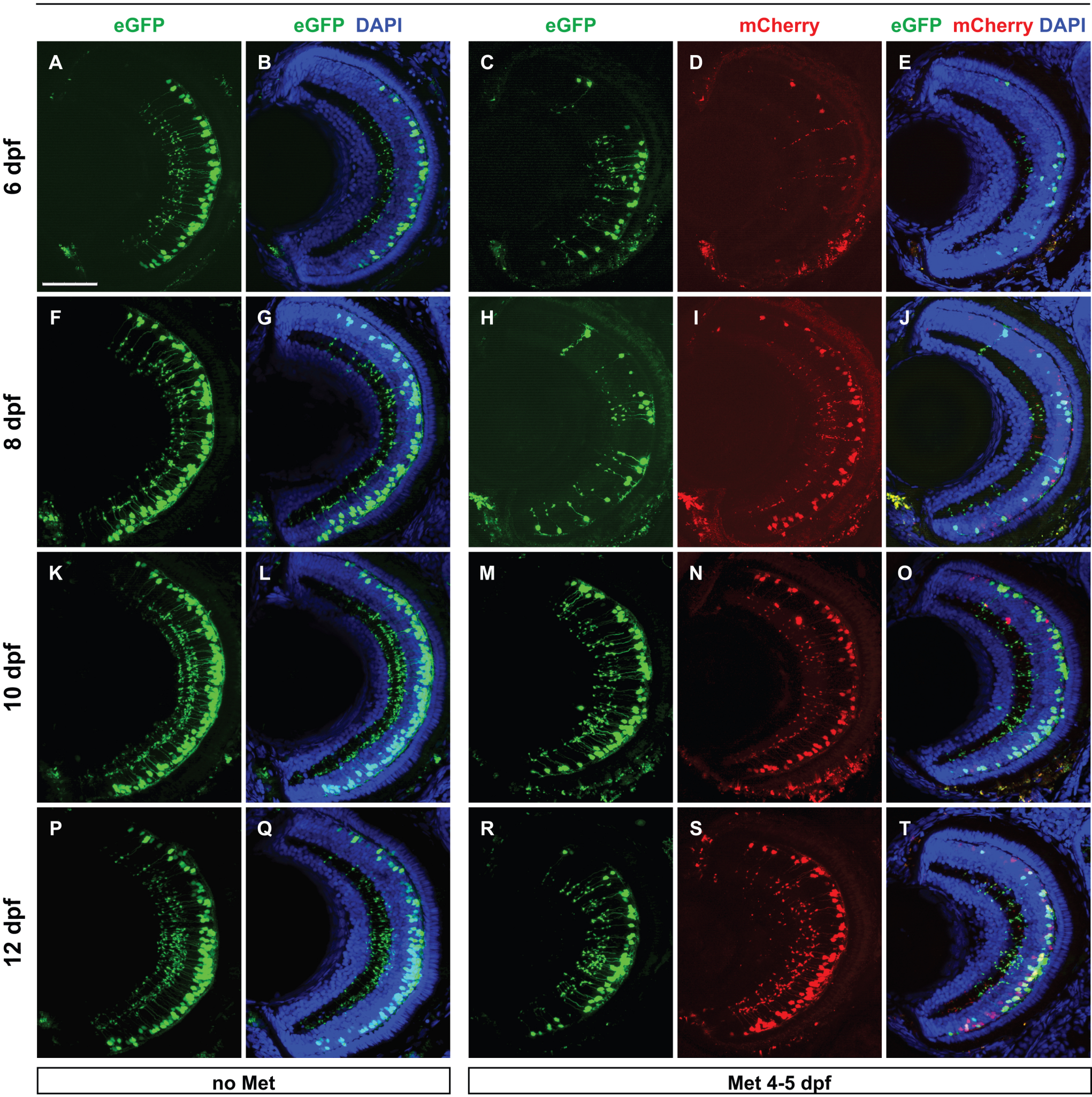

Supplement: Additional file 2 — Figure S2. Retina recovery in larval progeny from the xfz3 × UAS:nfsB-mCherry mating. Retinal cryosections were obtained from larvae at different time points during a period of seven days following removal of Met at 5 dpf. The left panel (labelled 'no Met' at the top) show confocal images of retina cross-sections from untreated siblings expressing only Gal4-VP16/eGFP. The right panel (labelled 'Met 4–5 dpf') show confocal images of retina cross-sections from NTR-mCherry expressing larvae following removal of Met. The different time points and corresponding larval stages are: (C-E) 1 day post-treatment (6 dpf); (H-J) 3 days (8 dpf); (M-O) 5 days (10 dpf); (R-T) 7 days (12 dpf). The different types of fluorescence are indicated at the top. Scale bar: 50 μm. [file 1471-2202-10-107-S2.pdf]

6 dpf

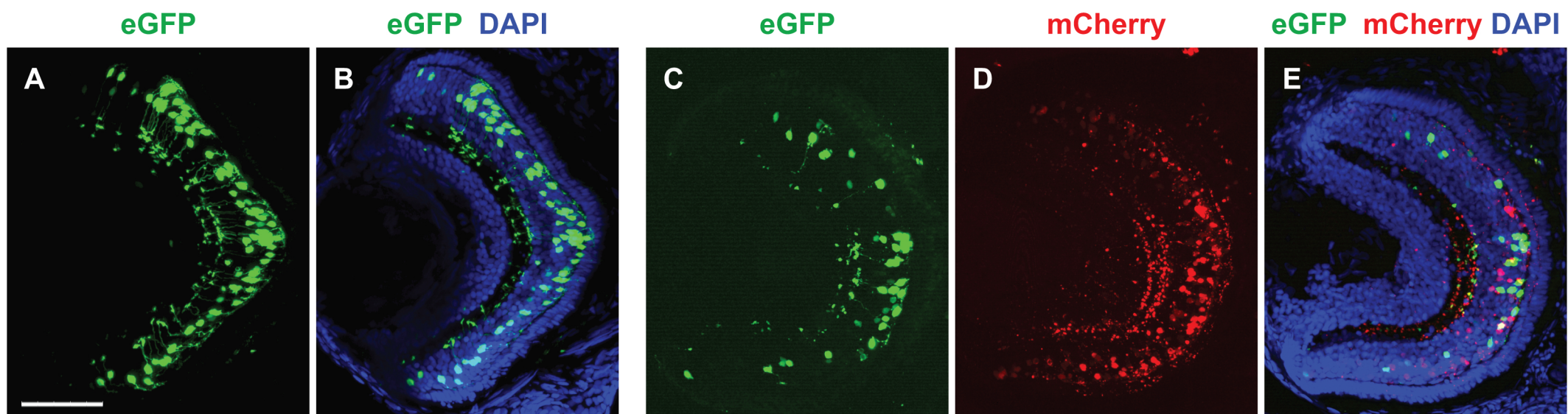

8 dpf

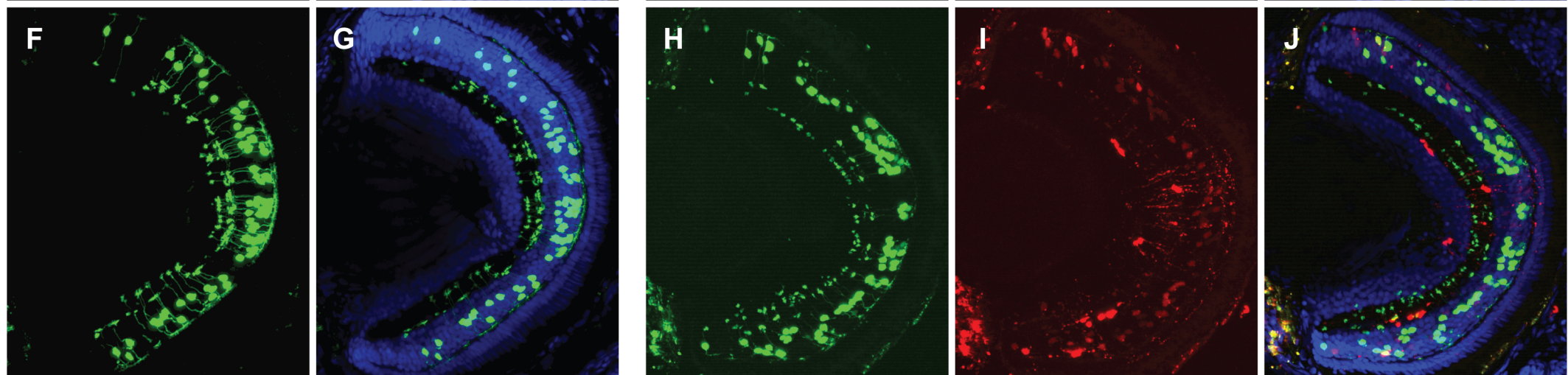

10 dpf

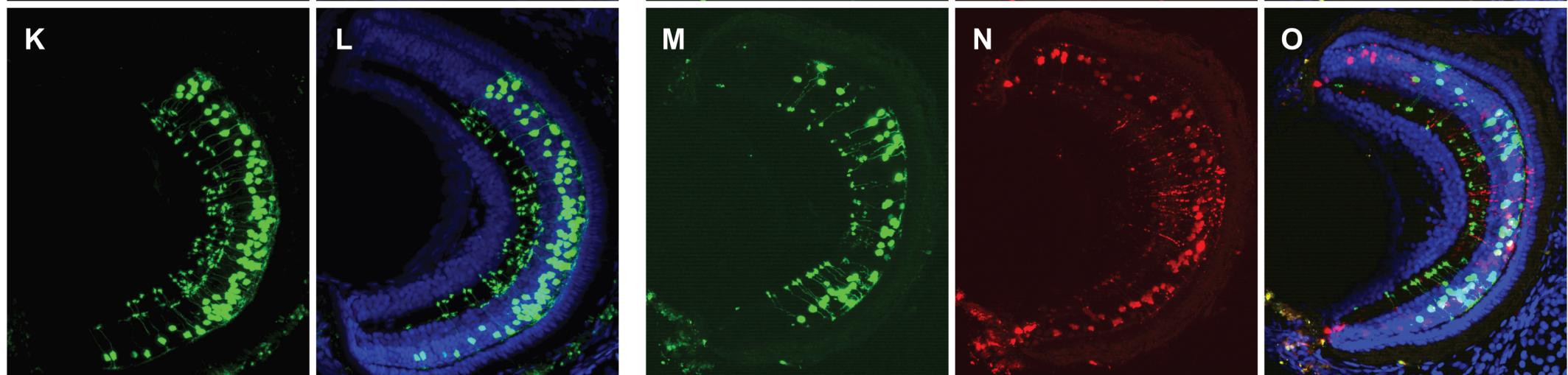

12 dpf

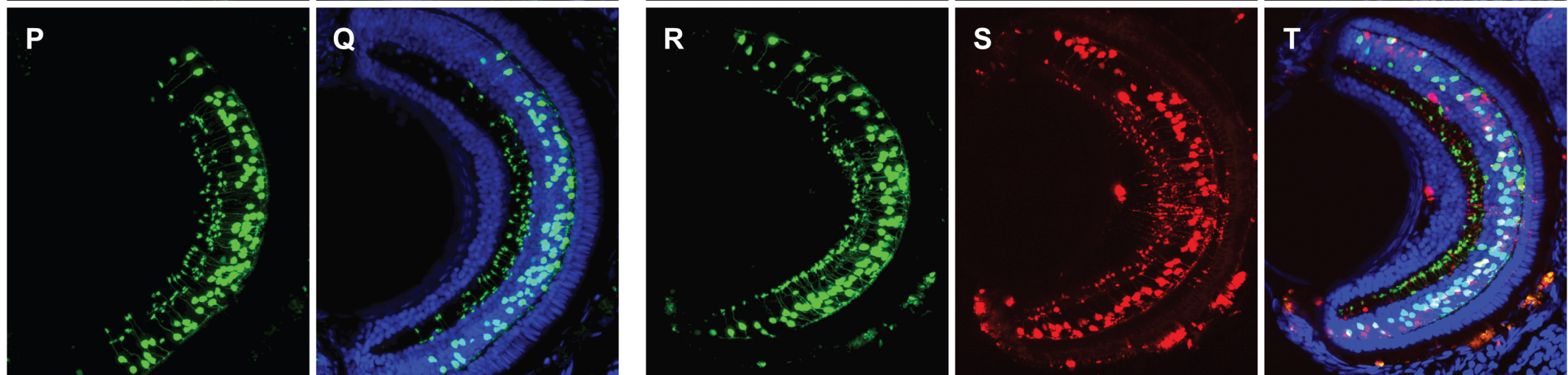

no Met

Met 4-5 dpf

Supplement: Additional file 3 — Figure S3. Retina recovery in larval progeny from the xfz43 × UAS:nfsB-mCherry mating. Confocal images of retina cross-sections from different stages of untreated siblings, which express only Gal4-VP16/eGFP (left panel), and Met treated larvae expressing NTR-mCherry (right panel) are arranged in the same way as described in Additional file 2: Figure S2. Scale bar: 50 μm. [file 1471-2202-10-107-S3.pdf]

# *xfz43* X *UAS:nfsB-mCherry*

**A**

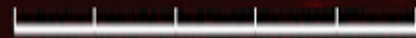

**Met -**

**B**

**Met +**

Supplement: Additional file 4 — Figure S4. Apoptotic cells in retina of prodrug treated larvae from the xfz43 × UAS:nfsB-mCherry mating. (A, B) Higher magnifications are shown for areas from Figure 7L, O, respectively. Retina from Met treated larva (B) show rounded NTR-mCherry labelled cells lacking axons. The axon termini in both the IPL and OPL (arrowheads) are also disintegrating. Scale bar: 25 μm. [file 1471-2202-10-107-S4.pdf]
