# Supplementary material for: Labelling and targeted ablation of specific bipolar cell types in the zebrafish retina
Source: BMC Neurosci. 2009 Aug 27;10:107. doi: 10.1186/1471-2202-10-107 (PMC3224687; doi:10.1186/1471-2202-10-107)
Supplement: Additional file 5 — Table S1. Genomic sequences flanking vector insertions. Zebrafish genomic sequences flanking the Tol2 vector insertions associated with retinal expression of Gal4-VP16/eGFP in the two transgenic lines. Tol2 vector sequences are underlined and coloured (right arm in red and left arm in blue). (Format: DOC). [file 1471-2202-10-107-S5.doc]

| line | Flanking sequences |
| --- | --- |
| *xfz3* | catgtatacttatttattttaaaaacatttgaaacaacaacaaagtatttactcaactgttgttgttattggctgaagaataaagtctttgttgcgttggccatctgactgcagctcatttcagatcctcgcgtcgcacaatcacgttttgtacctga cagaggtgtaaaaagtactcaaaaattttactcaagtgaaagtaca …  aaaatccccaaaaataatacttaagtacagtaatcaagtaaaattactcaagtactttacacctctg gtacctgattacatcacagcttgatctgcatgaaaagctgaaatcctgttggagggaatgcatggcagctttatacaatgagggattgttttcaaccaaagaccaccattggctgtaacaggggtcaaagttattcagtgtaagaggataaagtataaaaacccaaaaactcaagactgcaggaaaaaaacgtcgacggaagcagctgaaatcacagataacagtgaatacagcagtcatggtcagccggtgagtgactctattattttgatttattattttgcattgcaatatttggacataagtcagaattatcagccctcctgttatcataatatttgacta |
| *xfz43* | cacgtggccagacacagctt cagaggtgtaaaaagtactcaaaaattttactcaagtgaaagtaca …  aaaatccccaaaaataatacttaagtacagtaatcaagtaaaattactcaagtactttacacctctg  cacagcttggccactctcctgccagaaaatagcattttggacagtgtcccgagaagtccgttagcgtctaatctggggtctctcatctcccaggggaccacgcgctaaaccagggtgaagcccggaaaataaagacaaatgctctgtgtcacccgccttgcgcctccagtaagagactctcctttcggcgctgcactctgtaggtttttaaaagcaaacagcggcgtgcgatttttcaaaacattattatgcacagagaggctgtgtgcccatg |

**Table S1.**
